# Two types of amyloidosis presenting in a single patient: a case series

**DOI:** 10.1038/s41408-019-0193-9

**Published:** 2019-03-05

**Authors:** M. Hasib Sidiqi, Ellen D. McPhail, Jason D. Theis, Surendra Dasari, Julie A. Vrana, Maria Eleni Drosou, Nelson Leung, Suzanne Hayman, S. Vincent Rajkumar, Rahma Warsame, Stephen M. Ansell, Morie A. Gertz, Martha Grogan, Angela Dispenzieri

**Affiliations:** 1 Division of Hematology, Department of Internal Medicine, Mayo Clinic Rochester, Rochester, MN USA; 2 Department of Laboratory Medicine and Pathology, Mayo Clinic Rochester, Rochester, MN USA; 3 Department of Health Sciences Research, Mayo Clinic Rochester, Rochester, MN USA; 4 Division of Nephrology, Department of Internal Medicine, Mayo Clinic Rochester, Rochester, MN USA; 5 Department of Cardiovascular Diseases, Mayo Clinic, Rochester, MN USA

## Abstract

The amyloidoses are a group of disorders with overlapping clinical presentations, characterized by aggregation and tissue deposition of misfolded proteins. The nature and source of the amyloidogenic protein determines therapy, therefore correct subtyping is critical to patient management. We report the clinicopathologic features of nine patients diagnosed with two amyloid types confirmed by liquid chromatography-coupled tandem mass spectrometry. The most common types were transthyrethin (*n* = 9) and immunoglobulin-derived (*n* = 7). Two patients did not have immunoglobulin-derived amyloidosis despite the presence of a monoclonal gammopathy. Eight patients were diagnosed with two types concurrently, and one patient had an 11-year interval between diagnoses. Histopathological distribution of amyloid was variable with vascular, interstitial, and periosteal deposits seen. Identification of a second type was incidental in seven patients, but led to genetic counselling in one patient and therapy directed at both amyloid subtypes in another. With longer survival of myeloma and AL amyloidosis patients and increasing prevalence of patients with wild-type transthyretin amyloidosis due to an aging population, the phenomenon of two amyloid types in a single patient will be encountered more frequently. In light of revolutionary new therapies for transthyretin amyloidosis (patisiran, tafamidis, and inotersen), recognition of dual amyloid types is highly clinically relevant.

## Introduction

The amyloidoses are a group of disorders that are characterized by tissue deposition of misfolded proteins that results in progressive organ damage and dysfunction^1^. Misfolding of the protein precursor generates insoluble toxic protein aggregates in a B-sheet fibrillar structure that can be identified on tissue biopsies on the basis of their apple-green birefringence under a polarized light microscope after staining with Congo red^2^. Identifying the nature and source of the amyloidogenic protein is critical given therapy varies significantly for the different types of amyloidosis. Amyloid typing has evolved over time from reliance on immunohistochemistry, a method with poor sensitivity and specificity, to the introduction in recent years of laser-capture microdissection with liquid chromatography-coupled tandem mass spectrometry (LC/MS), a technique that allows accurate typing of amyloid with a high level of sensitivity and specificity^3^.

Immunoglobulin light chain (AL) amyloidosis is the most commonly recognized type of systemic amyloidosis with a reported incidence of approximately 6–10 cases per million per year^4,5^. AL amyloidosis results from a neoplastic plasma cell or B-cell clone producing amyloidogenic light chain and commonly presents with cardiac, renal, or peripheral nerve involvement. Transthyretin amyloidosis (ATTR) amyloidosis is also common, although its incidence is difficult to estimate as it is often asymptomatic or has subclinical manifestations. However, at least 24 additional amyloidogenic proteins that are potentially pathogenic have been identified in humans, and although each syndrome has its own unique features, there is considerable overlap in clinical presentation. Proteinuria is seen in over 50% of patients with AL amyloidosis and is also the most common clinical presentation in patients with serum amyloid A protein (SAA) amyloidosis^6^. Amyloid cardiomyopathy caused by wild-type transthyretin (ATTRwt) amyloidosis may be difficult to distinguish clinically from that caused by AL amyloidosis, though features such as age or a discordance between the extent of myocardial thickening and the extent of symptoms may serve as clues.

In addition to overlapping clinical presentation, there is the potential for two different types of amyloidosis to co-exist in the same patient, a phenomenon rarely reported in the literature^7–9^. Correct typing in this setting is of utmost importance to ensure proper therapy. Herein, we report a case series of patients seen at the Mayo Clinic with a confirmed diagnosis of two different types of amyloidosis.

## Methods

After approval by the Mayo Clinic Institutional review board we conducted a retrospective review of all patients at the Mayo Clinic that were diagnosed with two different types of amyloidosis. Only patients that had a confirmed diagnosis of two amyloid types in at least two separate specimens by laser-capture microdissection with LC/MS performed on each specimen were included in the study. The LC/MS method for amyloid typing has been previously described. In brief, 3–4 Congo red-positive foci of formalin-fixed, paraffin embedded tissue blocks were laser microdissected and the dissected tissue was digested into tryptic peptides and analyzed by LC–MS^3^. Patients were identified from a prospectively maintained database. Laser microdissection images and LC/MS data on all patients were reviewed. Specimens were considered a pure amyloid type if there were numerous spectra of peptides corresponding to that amyloid type and a mean value of ≤5 spectra of peptides corresponding to other types. An amyloid type was judged to be “predominant” if there were numerous spectra of peptides corresponding to that amyloid type and a minor component but still >5 spectra of peptides corresponding to a second amyloid type. An amyloid type was judged to be hybrid (e.g., “hybrid AL/ATTR”) if there were relatively equal spectral counts for the two amyloid types.

## Results

Of 1094 patients who had LC/MS-proven amyloid in more than one specimen, we identified 9 patients who had two different amyloid types in at least two separate specimens. Patient characteristics are summarized in Table 1. Clinical presentation in the seven patients diagnosed with both types of amyloid during initial workup, included cardiovascular symptoms (three), proteinuria (two), gastrointestinal symptoms (one), and lymphadenopathy (one). Median age at initial diagnosis was 74 (range: 59–90), and 8/9 patients were male. Eight patients were confirmed to have a plasma cell neoplasm, including the two patients with no evidence of AL amyloid (Cases 1 and 2). Three patients (cases 1, 8, and 9) had a history of a plasma cell disorder preceding the diagnosis of amyloidosis by many years (2 with monoclonal gammopathy of undetermined significance (MGUS) and 1 with smoldering myeloma). Immunofixation (of serum and/or urine) was positive in 7/8 patients tested, including the two non-AL amyloid patients. (99m)Tc-pyrophosphate scintigraphy (PYP-SPECT) scan was positive in 2/3 patients that had the test performed.

**Table 1:** Summary of characteristics of cases

Case	Age	Gender	Clinical symptoms	Monoclonal protein isotype	FLC ratio	BMPCs	Troponin (ng/mL)	NT-proBNP (pg/mL)	PYP	PCD diagnosis
1	86	F	GIT	λ	0.08	6%	<0.01	364	NA	LC–MGUS
2	74	M	CVS	κ	9.75	5%	0.06	5338	Positive	LC–MGUS
3	84	M	Proteinuria	IgG λ	0.51	5%	0.03	2134	NA	AL
4	90	M	Postmortem	NA	NA	NA	NA	NA	NA	–
5	59	M	Proteinuria	IgD λ	0.04	10–15%	0.03	315	NA	AL
6*	59	M	Lymphadenopathy	IgM κ	78.1	5%	0.02	241	NA	AL
7	79	M	CVS	NA	NA	NA	0.05	5960	NA	AL
8	70	M	Myopathy	IgG κ	275	8%	0.02	93	Negative	AL
9	66	M	CVS	Triclonal	37.4	5%	43**	895	Positive	AL

^*^Underlying lymphoplasmacytic lymphoma. ^**^High sensitivity troponin T assay (ng/L). *ATTR* transthyretin amyloidosis, *AL* immunoglobulin light chain amyloidosis, *LC–MGUS* light chain monoclonal gammopathy of undetermined significance, *CVS* cardiovascular symptoms, *GIT* gastrointestinal symptoms, *FLC* free light chain, *NT-proBNP* N-terminal pro-brain natriuretic peptide, *F* female, *M* male, *BMPCs* bone marrow plasma cells, *NA* not available, *PYP* (99m)Tc-pyrophosphate scintigraphy, *PCD* plasma cell disorder

ATTR was one of the amyloid types in all nine cases, and in seven cases the second amyloid type was immunoglobulin-derived (AL; *n* = 6; immunoglobulin-associated; *n* = 1). In the remaining two cases the second amyloid type was SAA and insulin-derived (AIns), Table 2. The TTR protein sequence was normal by mass spectrometry-based proteomic analysis in all cases but one; the negative result was confirmed by gene sequencing in one case. In case 8, mass spectrometry-based proteomic analysis detected an amino acid abnormality in the transthyretin protein (valine-to-isoleucine substitution at position 122 (Val122Ile)); which was confirmed by sequencing of the TTR gene and indicates hereditary ATTR amyloidosis (ATTRm).

**Table 2:** Pathologic summary

Case	Tissue 1	Anatomic distribution	Amyloid biopsy 1	Tissue 2	Interval (months)	Anatomic distribution	Amyloid biopsy 2	Focus of therapy
1	Stomach	Interstitial = ATTR; Vascular = ATTR	ATTR	Fat	4	NA	SAA	Nil
2	Heart	Interstitial = ATTR	ATTR	Fat	0	NA	AIns	ATTR
3	Kidney	Vascular = AL	AL (λ)	BM	2	Periosteal fibrous tissue = ATTR	ATTR	AL
4	Heart	Vascular = ATTR	ATTR	Duodenum	0	Interstitial = A-IA	A-IA	Nil**
5	BM	Periosteal fibrous Tissue = AL & ATTR	Hybrid AL (λ) and ATTR	Fat	0	NA	AL (λ)	AL
6^	BM	Periosteal fibrous tissue = AL & ATTR	Hybrid AL (κ) and ATTR	Lymph node	0	Globular = AL predominant	AL (κ) predominant	AL
7	Heart	Vascular = AL; Interstitial = ATTR Predominant	AL (λ) and ATTR*	Kidney	4	Vascular = AL	AL (λ)	Nil***
8	BM	Periosteal fibrous tissue = AL	AL (κ)	BM	130	Periosteal fibrous tissue = AL and ATTR	Hybrid AL (κ) and ATTRm	AL
9^^	Fat	NA	AL (λ)	Heart	1	Vascular = AL and ATTR; interstitial = AL and ATTR	Hybrid AL(λ) and ATTR	AL and ATTR

^*^Amyloid subtype varied depending on anatomic distribution. See Fig. 1

^**^Amyloidoses were diagnosed postmortem

^***^Patient expired prior to institution of therapy

^^^Heart biopsy 6 years later demonstrated vascular involvement by AL (κ) predominant amyloid

^^^^Prostate biopsy from 4 years earlier was retrospectively analyzed for amyloid, demonstrating AL (λ) amyloid in a vascular distribution

*ATTR* transthyretin amyloidosis, *ATTRm* hereditary transthyretin amyloidosis, *AL* immunoglobulin light chain amyloidosis, *A-IA* immunoglobulin-associated amyloidosis, *SAA* serum amyloid A amyloidosis, *AIns* insulin-derived amyloidosis, *BM* bone marrow

In 8 cases, both amyloid types were diagnosed concurrently or during initial work up (within 4 months), and in the other case there was an 11-year interval between diagnoses. One of the concurrent cases (case 9) had a prostate biopsy from 4 years prior that was retrospectively analyzed for amyloid, demonstrating the longstanding presence of AL amyloid.

Based on the proteomic data of the tissues, the cases could be divided into two categories (Table 2). The first was comprised of four patients (Cases 1–4) who had two distinct amyloid types involving two separate anatomic sites, which were diagnosed concurrently or during initial work-up (within 4 months). Pure ATTR amyloid was identified in the heart (*n* = 2), bone marrow (*n* = 1), and stomach (*n* = 1). The other amyloid type was AL (kidney), immunoglobulin-associated (duodenum), SAA (fat aspirate), and AIns (fat aspirate). In the three cases in which the amyloid was identified premortem, the amyloid type that was identified second (SAA, AIns, and ATTR) was an incidental finding and did not affect clinical management.

**Fig. 1:**
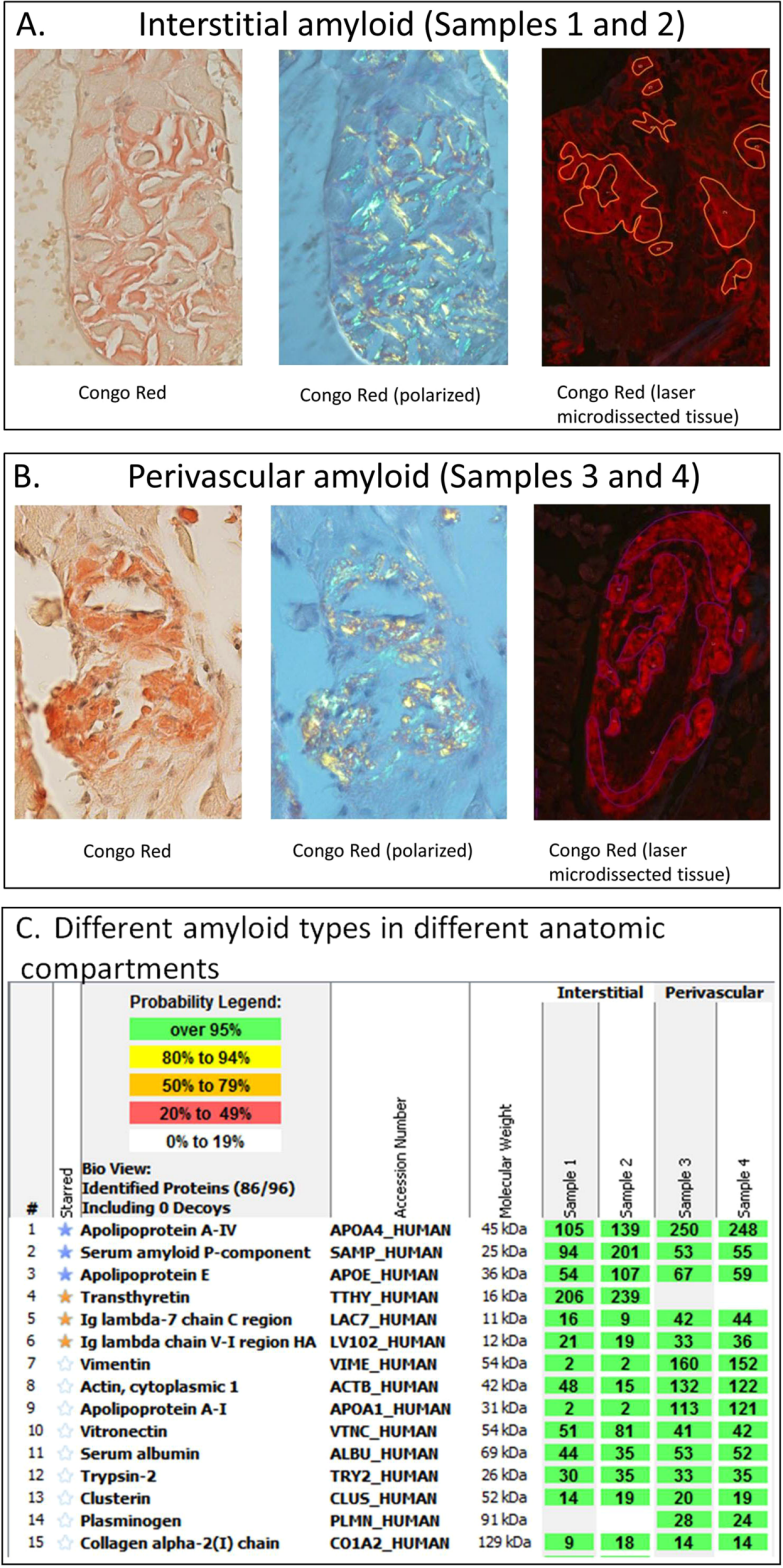
Two types of amyloid identified within different anatomic compartments on a single tissue sample. **a**, **b** Congo red stain of tissue from heart biopsy (Case 7) demonstrating interstitial (**a**) and vascular (**b**) amyloid deposits. **c** By LC/MS analysis, the interstitial amyloid deposits (Samples 1 and 2) contained a predominance of spectral counts for TTR peptides and a minor component of spectral counts for AL (λ) peptides (ATTR predominant), while the vascular amyloid deposits (Samples 3 and 4) contained elevated spectral counts exclusively for AL (λ) peptides (pure AL)

The second category was comprised of five patients (Cases 5–9) who had a combination of AL and ATTR amyloid detected in the same anatomic specimen. In all cases one specimen was hybrid AL/ATTR amyloid and other specimen(s) was/were AL amyloid (pure or predominant). In four cases, hybrid AL/ATTR involved a single-anatomic compartment within a single microdissection, and in the fifth case (case 7), two separate amyloid types were found in the same organ but in different anatomic compartments from separate microdissections (Fig. 1).

There were three cases with a significant time interval between biopsies. In case 6, the clinically significant amyloid (AL) was present in all biopsies and a prominent component of clinically insignificant ATTR amyloid was present only in the bone marrow. In cases 8 and 9, the amyloid type progressed from pure AL to hybrid AL/ATTR. The discovery of ATTR in addition to AL in case 9 led to institution of therapy targeting both subtypes of amyloidosis. In case 8, the diagnosis of ATTRm many years after a diagnosis of AL amyloidosis prompted genetic counselling but not intervention as the patient was not symptomatic from the ATTR amyloid. In the remaining cases, the second subtype of amyloid was either incidental and did not impact clinical management (ATTR; cases 5 and 6), or was not treated because the patient died prior to institution of any therapy (AL; case 7).

## Discussion

Our study reports on a rare but clinically important phenomenon, the occurrence of two different types of amyloidosis in individual patients. It is important to note that this is an uncommon phenomenon that we observed in <1% of cases in our laboratory. However, our study highlights a number of important points. First, this cohort demonstrates the need for accurate techniques for amyloid typing to ensure patients are not misdiagnosed or treated inappropriately. Prior to the availability of LC/MS, typing of amyloidosis relied on immunohistochemical methods, which have poor sensitivity and specificity owing to technical limitations and challenges^3,10,11^. LC/MS is a much more sensitive and specific technique allowing for accurate typing of the amyloidogenic protein^3,12,13^. The utility of accurate typing is highlighted by Case 2, a 75-year-old gentleman who 13 years postrenal allograft for diabetic nephropathy developed episodes of loss of consciousness with echocardiography revealing increased left ventricular thickness. Investigation for amyloidosis revealed an elevated free kappa light chain (35.6 mg/dL), 5% clonal plasma cells in the bone marrow, and the presence of amyloid in the fat aspirate. Given these results the suspicion for AL amyloidosis was high; however, PYP-SPECT scan was positive, suggesting ATTR which prompted further investigation with cardiac biopsy confirming ATTRwt by LC/MS. LC/MS of the fat aspirate revealed AIns, a form of iatrogenic amyloidosis related to insulin administration that is typically localized and benign. Definitive amyloid typing with LC/MS in this case avoided unnecessary and potentially toxic chemotherapy.

Second, performing definitive typing on all tissues that are biopsied and in which amyloid is identified can be clinically relevant. This may not always be necessary, particularly given guidelines for investigation of AL amyloidosis recommend concurrent bone marrow and fat aspirate biopsy^14^. In this setting performing LC/MS on both samples may be an inefficient use of resources; however, if the clinical picture is ambiguous, then LC/MS should be performed on all tissues available. Case 7, a 78-year-old male 10 years postkidney transplant for polycystic kidney disease who presented with cardiovascular symptoms, highlights this point. His initial cardiac biopsy showed ATTR-predominant amyloid involving the interstitium as well as pure AL amyloid involving the blood vessels, and his subsequent renal biopsy (for rising creatinine) demonstrated pure AL amyloidosis. This is another example where definitive typing by LC/MS has significant therapeutic implications.

Third, we identified two different categories of patients with two amyloid types: those with two distinct amyloid subtypes involving two separate anatomic sites, and those with a combination of AL and ATTR amyloid in the same microdissection. These cases highlight the importance of verifying the amyloid subtype in all involved specimens if the clinical picture is not clear, and they underscore yet another potential difficulty of correctly subtyping amyloid by immunohistochemical methods. Although the first category is diagnostically straightforward, the second category is quite intriguing raising the biologic question of in vivo seeding between two types of amyloidosis.

**Fig. 2:**
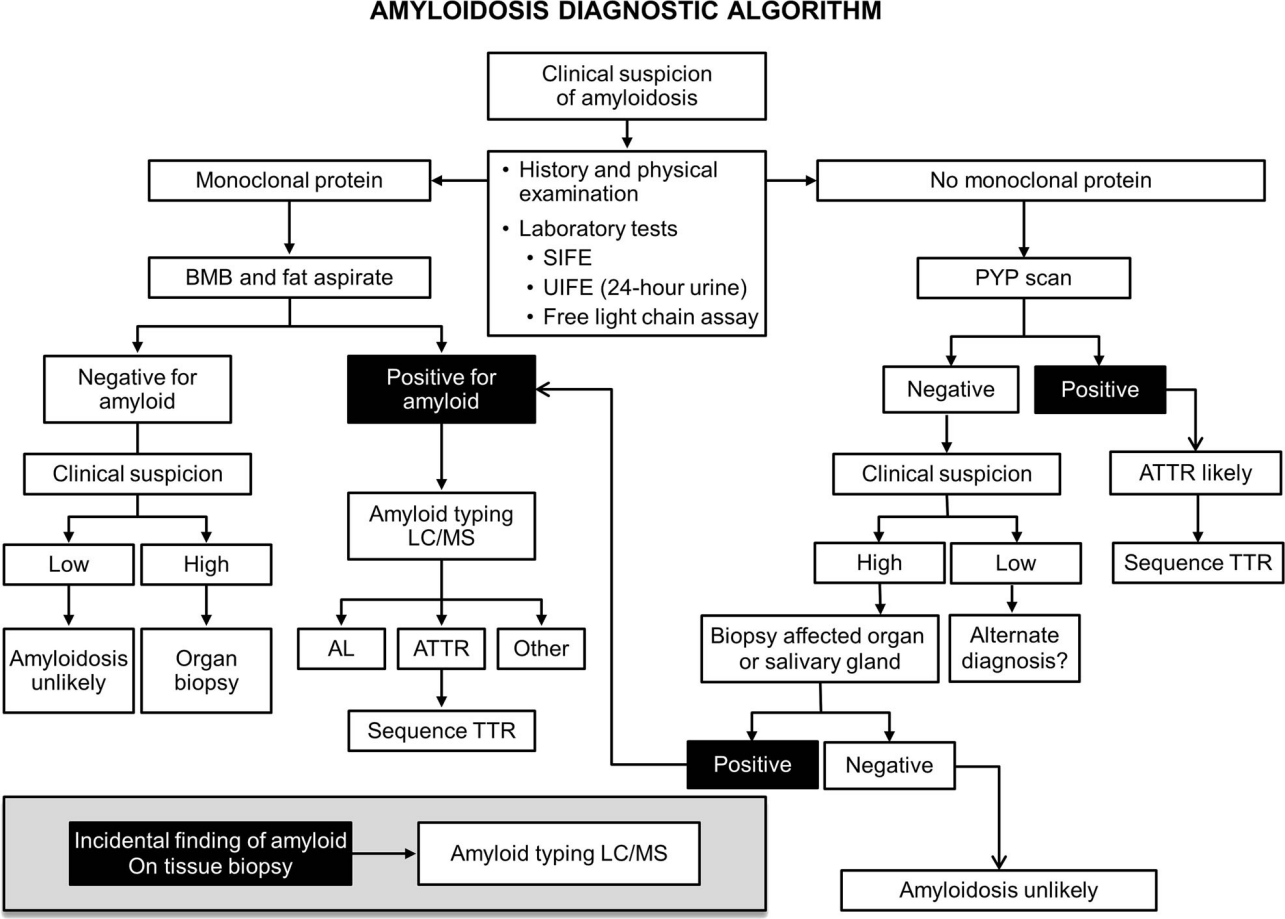
Amyloidosis diagnostic algorithm. ATTR transthyretin amyloidosis, AL immunoglobulin light chain amyloidosis, TTR transthyretin, BMB bone marrow biopsy, SIFE serum immunofixation electrophoresis, UIFE urine immunofixation electrophoresis, LC/MS liquid chromatography-coupled tandem mass spectrometry, PYP (99m)Tc-pyrophosphate scintigraphy

Fourth, a comprehensive assessment of patients suspected to have amyloidosis is important. Workup of patients with amyloidosis should incorporate a multidisciplinary team and requires a thorough history and physical examination, relevant laboratory tests, imaging studies, and histopathology review with appropriate typing techniques. PYP-SPECT has been identified as a valuable imaging modality to differentiate AL from ATTR cardiac amyloidosis^15^. It was performed in only three patients in our cohort in part due to the period in which these patients were diagnosed; it was critical in Case 2 where a positive test in a patient suspected to have AL amyloidosis prompted a cardiac biopsy, revealing ATTR. A complete assessment of these patients is particularly relevant given the varied clinical presentations of amyloidosis, ranging from asymptomatic incidental identification on tissue biopsy to multiorgan dysfunction from systemic amyloid deposition. An approach to investigation and diagnosis of patients suspected to have amyloidosis is summarized in Fig. 2.

Finally, it is important to remember this study included only patients who had amyloidosis with typing confirmed by LC/MS, a technology introduced in to our clinical practice from 2009 onwards. We do not typically type every biopsied specimen in a given patient, and as such we may be underestimating its occurrence. The situation is increasingly complex given the fact that MGUS, myeloma, and ATTR are all conditions of the elderly and that both myeloma and AL amyloidosis patients are living longer due to effective therapies^16,17^. The incidence of MGUS increases with age, from 3% of the population aged above 50–7.5% of those aged 85 and older^18^. The prevalence of ATTR amyloidosis also increases with age; one study of autopsies in patients aged 85 or older identifying ATTR in 25% of patients^19^. In patients with a pre morbid diagnosis of heart failure with preserved ejection fraction, ATTR was identified on autopsy in 32% of patients aged 75 or older^20^. Thus there is clear overlap in the demographics of patients that may be affected by AL and ATTR amyloidosis, increasing the likelihood of two diagnoses, each with a vastly different clinical course and treatment paradigm. With the emergence of treatments directed against ATTR (diflunisal, tafamadis, patisiran, and inotersen), finding ATTR is no longer just an academic exercise.

In conclusion, in light of the similarities in clinical presentation among the different types of amyloidosis, definitive typing of the amyloid is critical in establishing a diagnosis and instituting appropriate therapy for patients. Although rare, two different types of amyloidosis can occur in individual patients and clinicians need to consider this possibility and pursue further diagnostic testing when the clinical picture is incongruent with the established diagnosis.
